# Electrocatalytic CO_2_ Reduction to Methanol on Pt(111) Modified with a Pd Monolayer

**DOI:** 10.1021/acscatal.4c05442

**Published:** 2025-01-10

**Authors:** Aleksandra Wawrzyniak, Marc T. M. Koper

**Affiliations:** † Leiden Institute of Chemistry, Leiden University, Einsteinweg 55, 2333 CC Leiden, The Netherlands

**Keywords:** carbon dioxide reduction, methanol, electrocatalysis, palladium monolayer, single crystal

## Abstract

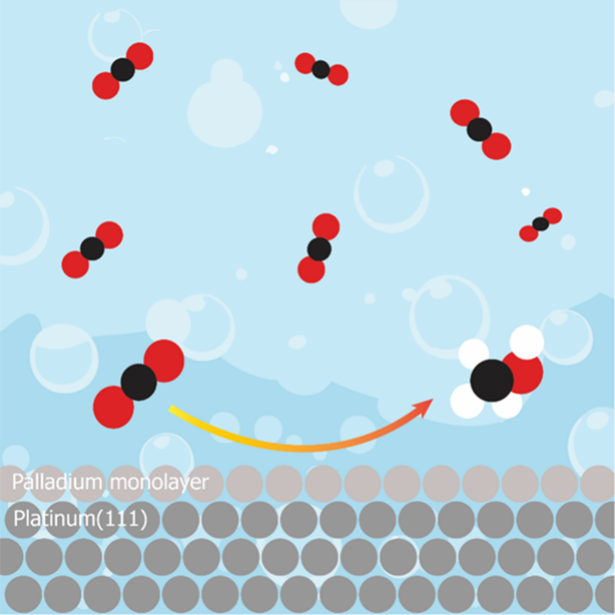

Electrochemical carbon dioxide (CO_2_) conversion to value-added, highly reduced chemicals such as methanol (CH_3_OH) is a promising possibility for producing renewable fuel and simultaneous CO_2_ recycling. However, this process remains a challenge, with only a few selective electrocatalysts known. Here, we present a study of a palladium monolayer on a platinum (111) single crystal (Pd_ML_/Pt(111)) as an electrocatalyst for CO_2_ conversion to CH_3_OH. A custom-made setup was employed in order to detect and quantify gaseous and liquid CO_2_ reduction products in sufficient concentrations despite the limitations of working with a single-crystalline electrode. Under ambient reaction conditions, a Faradaic efficiency (FE) of 1.5% at −0.9 V vs reversible hydrogen electrode (RHE) was obtained while using CO_2_ as the reactant. Other reaction intermediates, carbon monoxide (CO) and formaldehyde (HCHO) were subsequently used as reactants, leading to FEs of 1.8 and 2.5%, respectively, whereas formic acid is not reduced. The corresponding mechanism concluded from our work is compared to the literature. The electrocatalyst introduced here, with a highly well-defined structure for CO_2_ conversion to CH_3_OH, opens up possibilities for further catalytic explorations.

## 1 Introduction

Methanol is a vital platform molecule and currently mainly originates from nonrenewable fossil resources.^1^ It is broadly used in the industry to produce a variety of base chemicals and can be used as a (renewable) fuel.^2−5^ In order to reduce further CO_2_ output and to diminish the dependency on fossil fuels, it is essential that large-scale industrial production processes such as methanol production are replaced as much as possible with green-energy-based alternatives. Electrocatalytic production of methanol using CO_2_ and renewable electricity presents itself as a promising possibility.^6,7^ However, this process poses a challenge due to the multiple electron transfer steps (6e^–^) that have to take place in order to obtain highly reduced compounds such as methanol.^8^ Furthermore, steering the selectivity away from other possible C1 products (HCOO^–^, CH_4_) specifically to methanol adds to the complexity of the problem.

Only a limited number of electrocatalysts enabling CO_2_ conversion to methanol can be found in the literature. Originally reported by Kapusta and Hackerman,^9^ a molecular cobalt phthalocyanine (CoPc) catalyst has become the main electrocatalyst of interest for this particular reaction in the most recent years.^10−12^ Previously known for efficient CO_2_ to CO conversion,^13,14^ it was recently shown that very high Faradaic efficiencies (FEs) toward methanol of up to >80% can be obtained when CO is used as feedstock under 10 atm pressure and CoPc-NH_2_ supported on carbon nanotubes (CNTs) as a catalyst. Moreover, the carbon paper was additionally coated with a microporous layer (MPL) composed of carbon particles and fluoropolymers to enhance the CO transport within the catalyst layer.^15^ A thorough mechanistic study has been conducted by Ren et al., in which competition over adsorption sites between CO_2_ and CO has been established on CoPc/CNTs.^16^ Using in situ X-ray absorption spectroscopy (XAS), a varying adsorption configuration of *CO was discovered for CO_2_ and CO reduction, as well as a weaker stretching vibration of the C–O bond in CO reduction during Fourier transform infrared (FTIR) spectroscopy experiments. A significant increase (by almost 20%) in FE toward methanol was observed by employing a membrane electrode assembly (MEA) compared to an H-cell. Upon using a reactant mixture of 90% CO and 10% CO_2_, a notable decrease in FE_CH_3_OH_ was determined, leading the authors to conclude that CO_2_ binds stronger than CO. As a result, CO originating from CO_2_ reduction primarily desorbs instead of reacting further down the CH_3_OH pathway.

Apart from molecular catalysts, a few Pd-based catalysts have been reported to facilitate the production of methanol from CO_2_. It was shown that on hierarchical Pd/SnO_2_ nanosheets using an H-cell setup, CO_2_ was reduced to CH_3_OH, with a maximum FE of 54.8% at −0.24 V vs reversible hydrogen electrode (RHE).^17^ However, Pd nanosheets alone showed poor electrochemical activity. Another recently reported successful Pd-containing electrocatalyst was MnO_2_ nanosheets with Pd nanoparticles, achieving FE for methanol of 80.9% at −0.6 V vs RHE in a GDE-type membrane electrode assembly (MEA) electrolyzer setup.^18^ The Pd-MnO_2_ NSs were deposited on a Cu or Ni foam substrate. No production of methanol has been reported on pure Pd. In both cases, Pd, which by itself becomes easily poisoned with CO, was combined with a metal (Sn, Mn) that binds *CO very weakly. Combining Pd with such metals therefore appears to have opened up new reaction pathways for targeted CO_2_ conversion to CH_3_OH. Both bimetallic systems mentioned (Pd/SnO_2_, Pd/MnO_2_) show a synergistic effect of the metals, and the authors attribute the methanol production to such effects. However, it has not been explained what facilitates the mechanism toward methanol in the first place, since neither of those metals produces methanol by itself according to the discussed literature. It must be noted that there is a significant amount of convoluted experimental variables, such as surface structure, substrate material, and combination of metals. The contribution of all of these factors cannot be neglected when discussing the catalytic performance. As a consequence, the fundamental understanding of the mechanism and influential factors is still lacking, as all of the above-mentioned effects would have to first be disentangled to achieve that. Moreover, no stand-alone Pd-based catalyst toward methanol has been reported so far for CO_2_ conversion to CH_3_OH.

On the other hand, the experimental study of the pathways of CO_2_ reduction on Pd is hampered by the tendency of Pd to form bulk hydrides, which mask the surface processes.^19,20^ A Pd monolayer deposited on a Pt(111) single crystal (Pd_ML_/Pt(111)) is known to circumvent this bulk hydride formation (due to the absence of bulk Pd and Pt itself not forming bulk hydrides) while still having a similar reactivity as bulk Pd. A Pd monolayer is known from previous literature as a reasonably efficient and reversible catalyst for CO_2_ conversion to formate as well as for formic acid oxidation.^21,22^ Therefore, epitaxially grown Pd overlayers offer a straightforward way to study the electrocatalysis of Pd without significant experimental issues.^23^ Pd_ML_/Pt(111) has been previously studied by Chen et al. as a CO_2_RR-electrocatalyst.^21^ It was shown that CO poisoning on the Pd monolayer occurs at higher overpotentials than in the case of Pt(111), enabling formate production. For Pt, *CO covers around 70% of the surface at −0.5 V vs RHE, whereas for Pd_ML_, such coverage is reached at approximately −0.8 V vs RHE and remains constant at higher potentials. The amount of adsorbed CO was estimated from CO stripping voltammetry but gives no indication about the amount of produced and subsequently desorbed CO. No methanol has been reported in this work.

Moreover, quantifying the products of CO_2_ electrolysis, especially methanol, when using single-crystal-based catalysts such as Pd_ML_/Pt(111) has proven challenging due to the small electrode area, setup challenges, and the associated detection limits of analytical instruments. A previously used method for formate detection and quantification has been online high-performance liquid chromatography (HPLC), where the liquid sample is taken as close as possible from the crystalline surface at frequent time intervals.^21,22^ However, the distance from the sampling needle to the surface can be difficult to reproduce; the measured concentration will vary locally, depending on the precise position of the needle against the catalyst surface, and diffusion of the products to the bulk is neglected. Sampling from the meniscus also changes the composition and thickness of the meniscus, resulting in probable additional effects. Sampling from the bulk electrolyte circumvents all of these issues.

Moreover, proton nuclear magnetic resonance ^1^H NMR can be employed. For both analytical techniques, it is vital that the product concentration exceeds the detection limit of the measuring device. Regardless, accounting for 100% of Faradaic current in CO_2_ electrolysis while using single crystalline electrodes is a challenge in itself and requires a specialized setup.

In this study, we introduce, validate, and report (for the first time) that Pd_ML_/Pt(111) facilitates CO_2_ reduction to methanol with an FE of 1.45% at −0.9 V vs reversible hydrogen electrode (RHE) in KHCO_3_ during short-term electrolysis. Using CO as a reactant, which is a known intermediate from CO_2_ to CH_3_OH on other catalysts, gives only slightly increased FE_CH_3_OH_, 1.8% at −0.8 V vs RHE, while using formaldehyde (HCHO) resulted in nearly doubled FE_CH_3_OH_ of 2.5% at −0.7 V vs RHE. The influence of the intermediate concentrations was investigated, which further showcased the validity of the system and helped in elucidating the catalytic mechanism. Due to a specific H-cell design, liquid products were well above the detection limit for our analytical equipment of choice and could be successfully quantified. Lastly, as the presented catalyst Pd_ML_/Pt(111) is a well-defined and well-studied structure, it is a widely accessible surface for the conversion of CO_2_ to CH_3_OH, opening up many possibilities for further experimental and computational studies.

## 2 Materials and Methods

### 2.1 Chemicals

For the preparation of the electrolytes, the following chemicals were used: KHCO_3_ (99.95%, Sigma-Aldrich), H_2_SO_4_ (Merck Suprapur), PdSO_4_ (98%, Sigma-Aldrich), HCHO (16% methanol-free solution, Thermo Scientific), HCOOH (>98%, Sigma-Aldrich), and Milli-Q water (≥18.2 MΩ cm, TOC < 5 ppb). For the glass cleaning procedure, H_2_SO_4_ (95–98%, Sigma-Aldrich), H_2_O_2_ (35%, Merck), and KMnO_4_ (99%, Sigma-Aldrich) were used. The KHCO_3_ electrolyte was stored with Chelex (100 mM sodium form, Sigma-Aldrich). Ar (5.0 purity, Linde), CO (4.7 purity, Linde), and CO_2_ (4.5 purity, Linde) were used for purging the electrolytes. For the ^1^H NMR sample preparation, D_2_O was used (99.95%, Sigma-Aldrich).

### 2.2 Catalyst Surface Preparation

A Pt(111) single crystal (MaTeck, 10 mm diameter, 99.999%) was used as a substrate for the Pd monolayer working electrode. The single crystal was prepared prior to each experiment using the Clavilier method.^24,25^ The (111) surface structure was verified with blank cyclic voltammetry (CV) in 0.1 M H_2_SO_4_, followed by Pd deposition based on the method by Attard and Bannister.^26^ The Pt(111) single crystal was immersed into the acidic Pd^2+^-ion containing solution at 0.85 V vs the reversible hydrogen electrode (RHE), where no deposition occurred. The potential was then cycled between 0.85 and 0.1 V vs RHE. The deposition was terminated once the voltammetric peak at 0.23 V vs RHE did not increase further. Before each experiment, the cell was purged with Ar for at least 30 min. For the experiments where bare Pt(111) was used as a working electrode, CO annealing of Pt(111) was performed with subsequent CO stripping procedure in order to protect the surface from contaminants during the cell assembly.^27^ Subsequently, CO oxidation was performed before the electrolysis experiment.

### 2.3 Electrochemical CO_2_/CORR

#### 2.3.1 Setup

A custom-made 50.8 mm × 50.8 mm PEEK H-cell was used in a three-electrode setup with the catholyte chamber volume of 2 mL adapted for single-crystal electrodes (see Figures S1–S3 in the Supporting Information (SI) for the technical drawings). The CO_2_ gas was bubbled at the bottom of the cell through a PEEK frit (screening device) to enable fine bubble dispersion for at least 10 min before each experiment as well as throughout the experiments at a flow rate of 5 sccm. The gaseous flow rate was controlled with a mass flow controller (SLA5850, Brooks Instrument). An anion exchange membrane (AMVN Selemion, AGC) was employed to separate the cathode (prepared as described above) from the dimensionally stable anode (DSA, Magneto). A commercially available RHE (Mini-HydroFlex, Gaskatel) was used as a reference electrode and placed in the catholyte chamber. The catholyte chamber was coupled to an online gas chromatograph (GC 2014, Shimadzu) with an FID (Shincarbon column) and with a TCD detector (RTX-1 column). A gaseous sample was analyzed after 5, 19, and 32 min of electrolysis. At the end of each experiment, a liquid sample was taken from the catholyte chamber and analyzed by high-performance liquid chromatography (HPLC, Shimadzu) equipped with the Aminex HPX-87H column (Biorad) and by gas chromatography (Nexis GC 2030 with an AOC-30i autosampler, Shimadzu) with a SH-I-MS (Shimadzu) column. For additional liquid sample analysis to confirm the identity of methanol, ^1^H NMR was employed (Bruker AV-600). The ^1^H NMR sample composition was 450 μL of aqueous sample (postelectrolysis) and 50 μL of D_2_O.

#### 2.3.2 Electrochemical Methods

All glass and PEEK cells were cleaned in an acidic potassium permanganate solution overnight. The following day, the permanganate solution was drained, and the glass and PEEK parts were rinsed five times with Milli-Q-water. Afterward, they were immersed in dilute H_2_SO_4_ and H_2_O_2_ mixture and rinsed again multiple times with Milli-Q water. As the last step, all glass and the PEEK cell were boiled five times in Milli-Q water and rinsed repetitively.

For the voltammetry experiments, a Biologic SP-500 potentiostat was employed. Cyclic voltammetry (CV) was performed to characterize the working electrode surface and conduct electrochemical deposition. For electrolysis experiments, chronoamperometry (CA) was performed with an IviumStat potentiostat (Ivium Technologies), where a chosen potential was applied for 32 min. The electrolyte used in all electrolysis experiments was 0.1 M KHCO_3_. Prior to electrolysis, the Ohmic resistance was measured by electrochemical impedance spectroscopy (EIS) at −0.05 V vs RHE, and 85% Ohmic drop compensation was performed for all CA measurements. Before each electrolysis experiment, the PEEK cell was purged with CO_2_ or Ar for at least 10 min, while for voltammetry experiments, the glass cells were purged with Ar for at least 30 min.

#### 2.3.3 Formaldehyde Experiments

1, 10, and 100 mM HCHO solutions were prepared from methanol-free stock solution (used as purchased) in Milli-Q water. 1.62 mL of 10 mM HCHO was added to 0.18 mL of 1 M KHCO_3_ directly in the H-cell, and simultaneously, a blank sample was made in a vial using the same amount of electrolyte. After 32 min of electrolysis, a sample was taken from the electrochemical cell. Directly afterward, both samples were slightly acidified at the same time using dilute H_2_SO_4_ to stop the Cannizarro reaction. To determine the amount of methanol produced in the Faradaic process, the amount of methanol in the blank sample was subtracted from the sample obtained after the electrolysis.

## 3 Results and Discussion

### 3.1 Surface Preparation and Characterization

**Figure 1:**
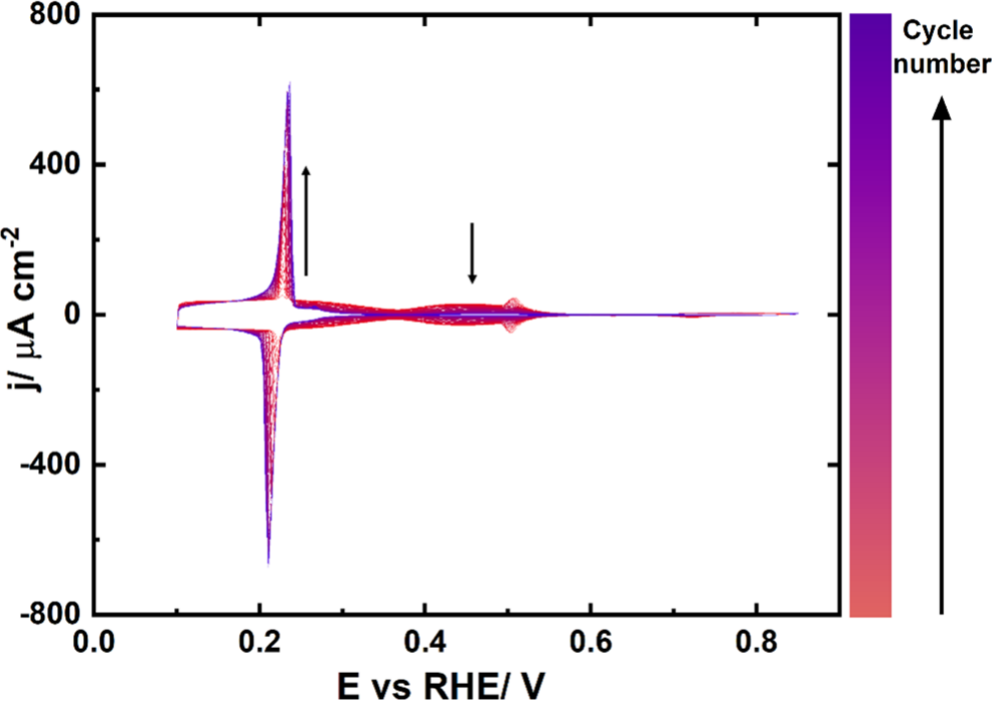
Pd electrodeposition process on the Pt(111) single crystal in 0.1 M H_2_SO_4_ at a scan rate of 50 mV/s. The total number of cycles was 117.

The electrochemistry of the Pd_ML_/Pt(111) single-crystal electrode has been well characterized and described in previous literature.^23,28^ As seen in Figure 1, Pd deposition requires several cycles, and the final deposited amount is a function of the concentration of the Pd^2+^ solution and the number of cycles. In Figure 1, a concentration of <0.01 mM Pd^2+^ was used in order to obtain a slow, gradual process, which can be followed precisely. The evolution of the deposition is first followed by observing the attenuation of the butterfly peak at 0.5 V vs RHE in sulfuric acid, characteristic for Pt(111), and the simultaneous increase of the peak characteristic for sulfate adsorption on Pd_ML_/Pt(111) at 0.23 V vs RHE. The deposition is interrupted once an additional shoulder, assigned to the start of the bilayer formation,^29^ arises at 0.28 V vs RHE. Further, the stability of the Pd monolayer was confirmed by comparing cyclic voltammograms in 0.1 M H_2_SO_4_ before and after the electrolysis (see Figure S4). The CVs overlap entirely.

### 3.2 CO_2_RR on Pd_ML_/Pt(111)

**Figure 2:**
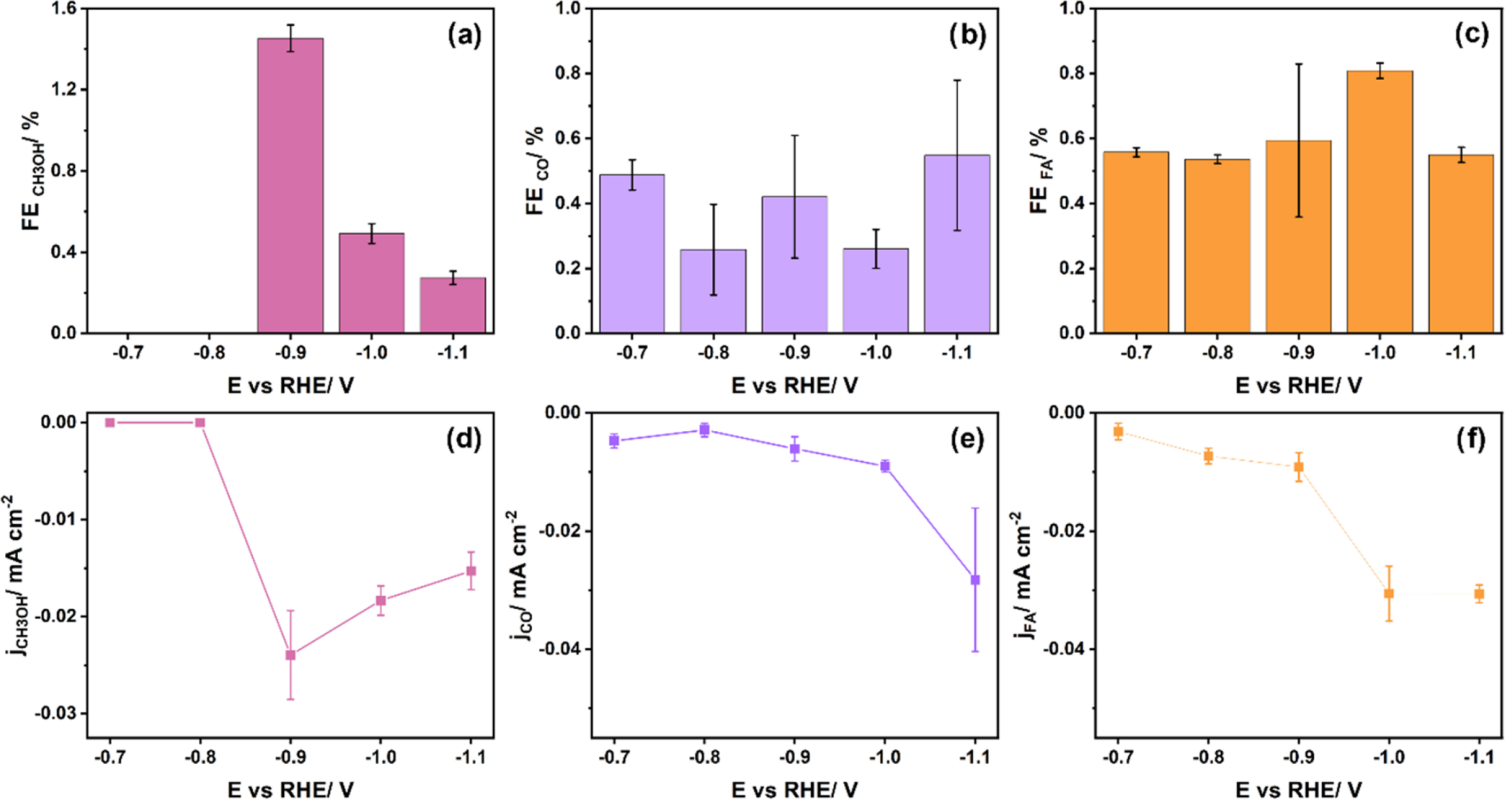
(a–c) Faradaic efficiencies for methanol, formic acid, and CO, respectively, in CO_2_RR. (d–f) Corresponding partial current densities. The reaction was performed under ambient conditions in 0.1 M KHCO_3_ (pH 7), and the duration of the electrolysis was 32 min. The remaining Faradaic efficiency is related to hydrogen evolution (Figure S8 in the SI).

CO_2_RR experiments were performed on the Pd_ML_/Pt(111) electrode under ambient conditions in 0.1 M KHCO_3_. In Figure S5, the corresponding *j*–*t*-curves can be found for all five investigated potentials. A decrease in current density has been observed for all potentials in the beginning of the electrolysis, and the decrease was always followed by the stabilization of the current density values. Figure 2a–c shows the Faradaic efficiencies for methanol, CO, and formic acid, respectively, as a function of potential, while Figure 2d,e shows corresponding partial current densities. Methanol was detected in the liquid sample using ^1^H NMR at −0.7 and −0.8 V vs RHE (see Figure S6 in the Supporting Information); however, it was below the detection limit of the GC used for liquid sample analysis. We conclude that the partial current density *j*_CH_3_OH_ at −0.7 and −0.8 V vs RHE must be smaller than −0.015 mA cm^–2^, which is the lowest partial current density in the graph. The highest FE toward methanol of 1.45% was reached at −0.9 V vs RHE, decreasing with more negative potential. The partial current density toward CH_3_OH most likely increased until −0.9 V vs RHE and decreased with increasing potential. The other products were CO, formate, and H_2_. Formaldehyde was not observed as an intermediate as presumably its concentration is too low because it is quickly reduced further to methanol. To exclude any contribution of formate reduction to the methanol formation, HCOOH (1 and 10 mM) electrolysis was conducted at −0.7 V vs RHE. While a significant enhancement of the HER was observed, no methanol was detected by means of the GC and ^1^H NMR (for the ^1^H NMR spectrum, see Figure S7). For (dissolved) CO, there is no clear potential dependency in the chosen potential range and all FE remain <1%. In the case of formate, the highest FE of 0.81% was reached at −1.0 V vs RHE. Partial current densities increase consistently with more negative potential for both CO and formate (Figure 2e,f). When comparing these results to the work by Chen et al.,^21^ a few differences become clear and possibly stem from different kinetics due to varying types of experiments in both cases. In this study, we employ chronoamperometry (constant potential for 32 min), whereas, in the aforementioned literature, linear sweep voltammetry at 1 mV/s was performed. It is crucial to underline that there is a significant difference between adsorbed CO that can be oxidized from the surface by cycling to positive potentials and subsequently desorbed from the CO that we detect in the gas phase. In the experiments by Chen et al., the CO coverage on Pd_ML_ reaches a plateau from −0.6 V vs RHE. Our observations align with this data, since we do not observe a clear trend in CO production for the investigated potential window. In the case of formate production, in the described study, the highest concentration of formate occurred at −0.6 V vs RHE. In our case, we did not perform experiments at that potential. Between −0.7 and −1 V vs RHE, it was observed by Chen et al. that the concentration of formate decreases, while here, we see a slightly increasing trend regarding the FE with the more negative potential. This discrepancy can be attributed to the varying electrochemical methods, as described above.

To compare our results to another benchmark research: in the study done by Boutin et al., in which CoPc on a GDE was employed as an electrocatalyst for CO_2_ reduction, the corresponding FE for methanol was 0.3% at −0.88 V vs RHE,^10^ under analogous reaction conditions. In alkaline pH (13), using CO as a reactant, FE_CH_3_OH_ of 14.3% was measured.

The catalytic properties of the Pd_ML_/Pt(111) surface were further studied by using known intermediates as reactants; in the first case, we used CO as feed gas, and further, HCHO was added to the electrolyte.^30^

### 3.3 Intermediate Conversion: CORR on Pd_ML_/Pt(111)

**Figure 3:**
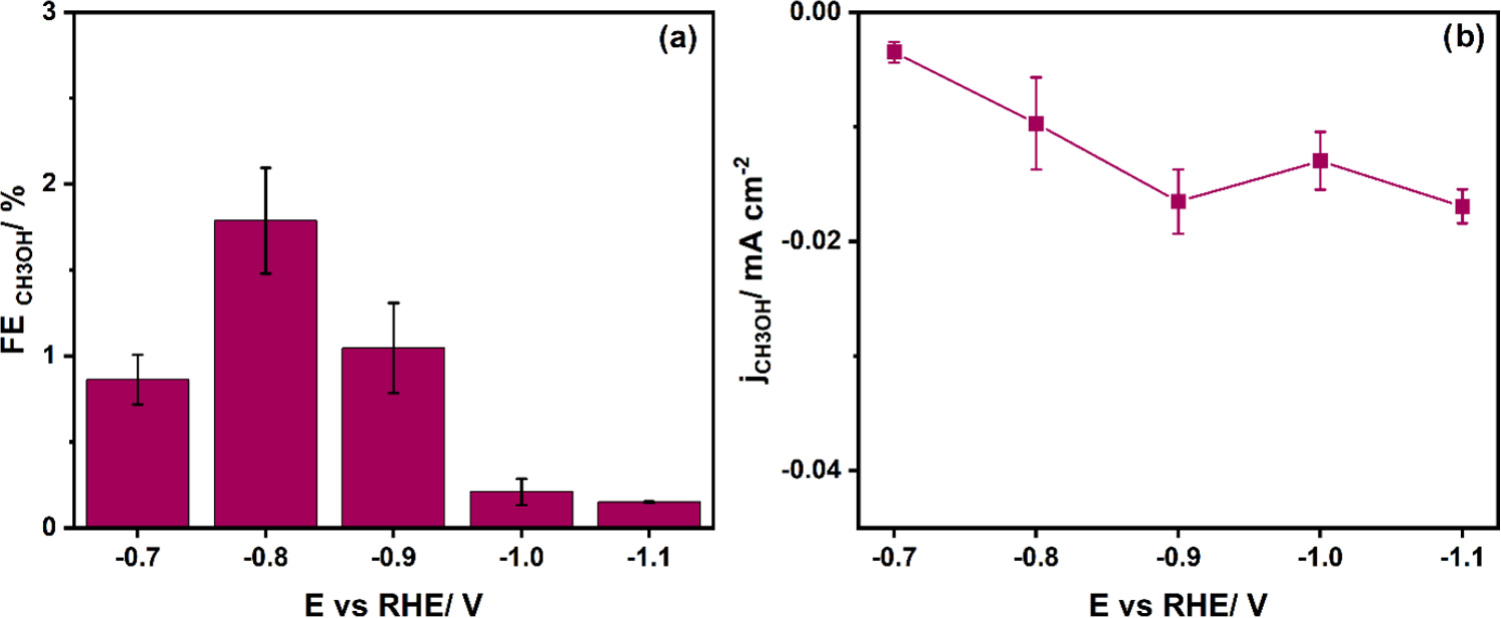
(a) Faradaic efficiency in the CORR for methanol. (b) Corresponding partial current densities. The reaction was performed under ambient conditions in 0.1 M KHCO_3_ (pH 9), and the duration of the electrolysis was 32 min.

CO reduction experiments were performed on a palladium monolayer in 0.1 M KHCO_3_. The only reaction products measured were methanol and hydrogen. No formate was detected in the sample analyzed with ^1^H NMR at −0.8 V vs RHE (see Figure S8). Figure 3a shows the Faradaic efficiency toward methanol in the same potential range as in CO_2_RR experiments. The FE_CH_3_OH_ reaches an optimum value of 1.78% at −0.8 V vs RHE. With a more negative potential, the FE_CH_3_OH_ decreases. In Figure 3b, we show that the partial current density to methanol increases slightly with more negative potential, in contrast to the results with CO_2_RR. For corresponding HER data, see Figure S9 in the Supporting Information.

Finally, as no formate was detected during CO reduction, we conclude the absence of the Cannizzaro reaction at the electrode interface,^31^ i.e., there is no base-promoted disproportionation of formaldehyde.

### 3.4 HCHO Reduction on Pd_ML_/Pt(111)

**Figure 4:**
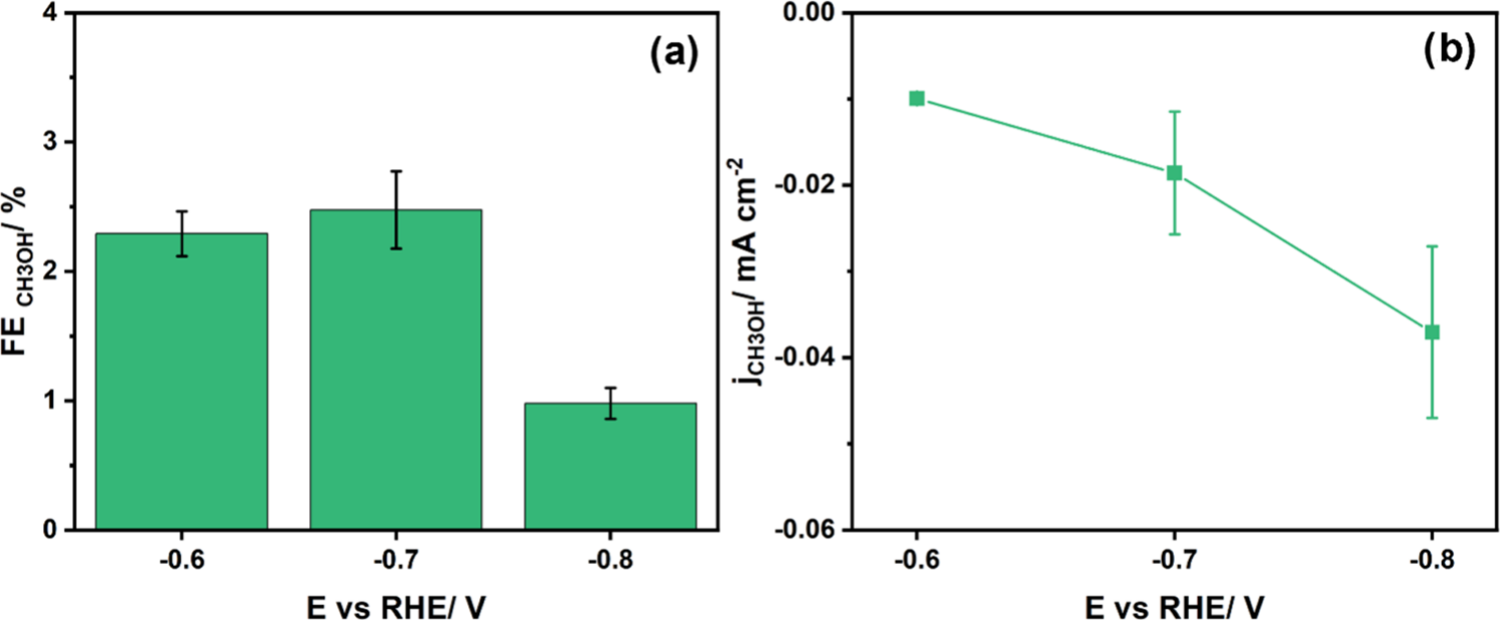
(a) Faradaic efficiency in HCHORR for methanol and (b) corresponding partial current densities. The reaction was performed under ambient conditions in 0.1 M KHCO_3_ (pH 9) with the addition of HCHO (10 mM) in an Ar atmosphere. The duration of the electrolysis was 32 min.

Formaldehyde HCHO is a known intermediate in CO_2_RR and CORR to CH_3_OH.^30^ Therefore, formaldehyde reduction was conducted in KHCO_3_ under an Ar atmosphere, with H_2_ and MeOH as the only products. In Figure 4a, the maximum obtained FE for methanol is 2.46% at −0.7 V vs RHE with a partial current density of −0.02 mA/cm^2^ (Figure 4b). Methanol is produced in significant quantities already at lower potentials compared with the CO_2_RR and CORR experiments. At potentials more negative than −0.7 V vs RHE, the rate of HER increases (Figure S10), and the methanol production decreases. However, compared to a study by Boutin et al. on CoPc, we obtained a lower FE_CH_3_OH_ from HCHO (they obtained 18% in pH 13 at −0.56 V vs RHE).^10^

### 3.5 Influence of Intermediate Concentration

**Figure 5:**
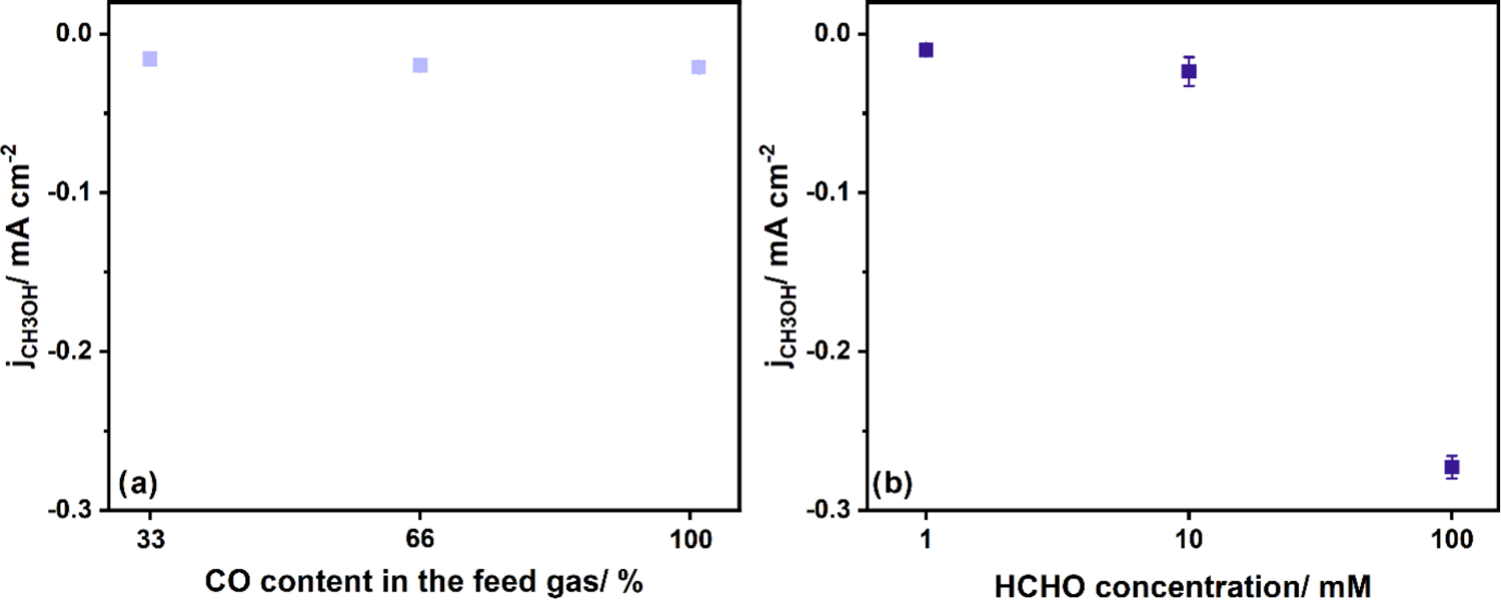
(a) Partial current density for MeOH as a function of the CO content in the feed gas stream (Ar). (b) Partial current density for MeOH as a function of HCHO concentration in the electrolyte. The reaction was performed under ambient conditions in 0.1 M KHCO_3_, and the duration of the electrolysis was 32 min.

To understand which reaction step is rate-determining, we performed experiments with varying the CO concentration in the feed gas flow while keeping the total flow constant. CO made up 33, 66, or 100% of the gas flow, while the remaining part consisted exclusively of argon. As can be observed from Figure 5a, the CO concentration has no significant impact on *j*_MeOH_. As Pd_ML_/Pt(111) has reached the maximum CO coverage between 0.7 and 0.8 ML under these conditions,^21^ this implies that only the adsorbed CO can be further converted to methanol. From our previous work, we can also conclude that there is no difference in the CO coverage for an electrode exposed to either CO_2_ or CO.^21^ In both cases, the electrode reached its maximum coverage. Therefore, CO and CO_2_ reduction to methanol takes place on a CO-modified electrode.

Furthermore, experiments using three different HCHO concentrations (1, 10, and 100 mM) were conducted. In Figure 5b, a clear dependency can be seen between the HCHO concentration and partial current density *j*_MeOH_, in which *j*_MeOH_ increases significantly with increasing HCHO concentration. This result implies that the amount of produced methanol is very strongly dependent on the amount of HCHO and that *CO to *CHO would be the likely rate-determining step (RDS) in the CO reduction. This would agree with the work by Li et al., who proposed a mechanism for CORR to CH_3_OH on CoPc; *CHO is also most likely the product of a rate-determining step.^15^

### 3.6 CO_2_RR on Pt(111)

**Figure 6:**
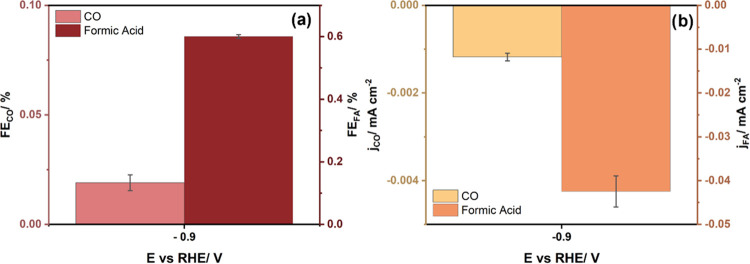
(a) Faradaic efficiencies toward CO and FA on Pt(111) in CO_2_RR. (b) Corresponding partial current densities. The reaction was performed under ambient conditions in 0.1 M KHCO_3_ (pH 7), and the duration of the electrolysis was 32 min.

CO_2_RR was also conducted on Pt(111) single crystal at −0.9 V vs RHE, which was found to be the optimum potential for CO_2_RR on Pd_ML_/Pt(111), as a control experiment to confirm our ability to account for 100% of Faradaic efficiencies using the setup as well as detect and quantify minor reaction products. The products detected were CO with an FE of 0.02% as well as formate with an FE of 0.6%, as shown in Figure 6a. No methanol was detected. For corresponding partial current densities, see Figure 6b, and for HER data, Figure S11.

### 3.7 General Discussion and Conclusions

Here, we have shown for the first time how a Pd monolayer on Pt(111), Pd_ML_/Pt(111), serves as an electrocatalyst facilitating the conversion of CO_2_ to methanol, with a 1.45% FE at −0.9 V vs RHE from CO_2_. The FE to CH_3_OH does not increase significantly in the CORR (1.78% at −0.8 V vs RHE). However, when using HCHO as a starting compound, a larger FE_CH_3_OH_ of 2.46% at −0.7 V vs RHE is obtained at a lower potential compared to that of the CO_2_RR. Moreover, a very clear dependency has been established between the HCHO concentration and MeOH production, whereas the CO concentration in the feed gas does not impact the MeOH production. Based on these observations, it can be concluded that *CO to *CHO would likely be the rate-determining step of the CORR. Because our study involved a single-crystal electrode, we employed a special electrochemical setup, which allowed us to account for 100% of the Faradaic efficiency in every experiment. In the case of CO_2_ conversion, methanol, formate, CO, and H_2_ are the only products of the reaction; no conclusions about the impact of the Cannizzaro reaction can be drawn in the case of the CO_2_RR as the amount of formate is more than 10 times the amount of methanol. On the other hand, upon performing CO reduction, no formate was detected, which excludes the presence of the Cannizzaro reaction at the interface under these conditions, confirming that Pd_ML_/Pt(111) facilitates the mechanism of CO_2_ conversion to methanol.

It is important to note that the substrate on which the Pd monolayer is deposited plays a significant role in its reactivity. In previous work by Kortlever et al., it has been shown that a Pd monolayer on gold foil facilitates C1–C3 hydrocarbon production.^32^ With an increasing Pd thin film thickness, longer chains up to C5 were obtained. Trace amounts of MeOH were detected using ^1^H NMR; however, they have not been quantified. Furthermore, a thin Pd film on silver has been investigated under analogous CO_2_RR conditions, and only methane and ethylene were detected.^32^ In our study of Pd_ML_/Pt(111), no hydrocarbons were detected, indicating obvious differences in the reactivity of these systems. Due to the crucial impact of the substrate material on the reactivity of the catalyst, this subject definitely warrants further investigation in the future, both experimentally and theoretically.

The mechanism for methanol formation is very similar to the mechanism previously concluded for CO_2_RR to methanol on a molecular Co-based catalyst.^10^ CO_2_ is reduced to CO and, subsequently, to formaldehyde and methanol. The rate-determining step appears to be the conversion of (adsorbed) CO to formaldehyde, similar to previous conclusions by Li et al.^15^ Formic acid is a side product of the CO_2_RR but is not converted further. Remarkably, this pathway is operative on the Pd_ML_/Pt(111) electrode but not on the Pt(111) electrode. We have previously argued that hydride formation is crucial for the special hydrogenation capability of the Pd-modified electrode vs pure platinum.^33^ Also, the reaction takes place on a surface that is (almost) fully covered by adsorbed CO. This explains why hydrogen still is the main product, but any small remaining differences in the CO adlayer on the Pd_ML_/Pt(111) electrode vs the Pt(111) electrode may also play a role in the hydrogenation capability of the surface. Future work will need to elucidate the role of the CO adlayer and how the hydrogen evolution reaction may be suppressed by optimization of the catalyst–electrolyte interface.

Finally, the Pd_ML_/Pt(111) electrode shows a FE_CH_3_OH_ via CO_2_ reduction almost 5 times higher than when using CoPc under analogous reaction conditions.^10^ Moreover, we were able to achieve such results using a single-crystalline electrode surface, while in the aforementioned study, a GDE was applied. In the case of CoPc, upon improvements and further research, FE toward methanol could be improved drastically from 0.3% in 2019 (Boutin et al.^10^) to 70–80% over the course of the past 2 years.^15^ The system described in our work already has a much higher starting point than CoPc originally and therefore could be very promising for further exploration, for instance, by modification with other metals. While it clearly still requires further study and optimization, such as HER suppression, the effect of the electrolyte, and the substrate, it opens up the unique opportunity of investigating well-defined, modified Pd-based electrocatalysts with improved activity and selectivity.

## Supporting Information Available

Cell design, NMR data, hydrogen Faradaic efficiencies, and cyclic voltammetry of Pd_ML_/Pt(111) (PDF)
