# Supplementary material for: Electrocatalytic CO2 Reduction to Methanol on Pt(111) Modified with a Pd Monolayer
Source: ACS Catal. 2025 Jan 10;15(3):1514–21. doi: 10.1021/acscatal.4c05442 (PMC11811924; doi:10.1021/acscatal.4c05442)
Supplement: Supplementary file 1 — cs4c05442_si_001.pdf [file cs4c05442_si_001.pdf]

Electrocatalytic $CO_{(2)}$ reduction to Methanol on Pt(111) modified with a Pd Monolayer

Aleksandra Wawrzyniak[1], Marc T. M. Koper[1,*]

[1] *Leiden Institute of Chemistry, Leiden University, Einsteinweg 55, 2333 CC Leiden, the Netherlands;*
**m.koper@lic.leidenuniv.nl*

Supplementary Information

Cell design	2
Cyclic voltammetry of Pd_{ML}/Pt(111)	4
Chronoamperometry curves	4
NMR data	5
HER data for $CO_{(2)}$RR on Pd_{ML}	7
HER data for HCHORR on Pd_{ML}	8
HER data for Pt(111)	8

Cell design

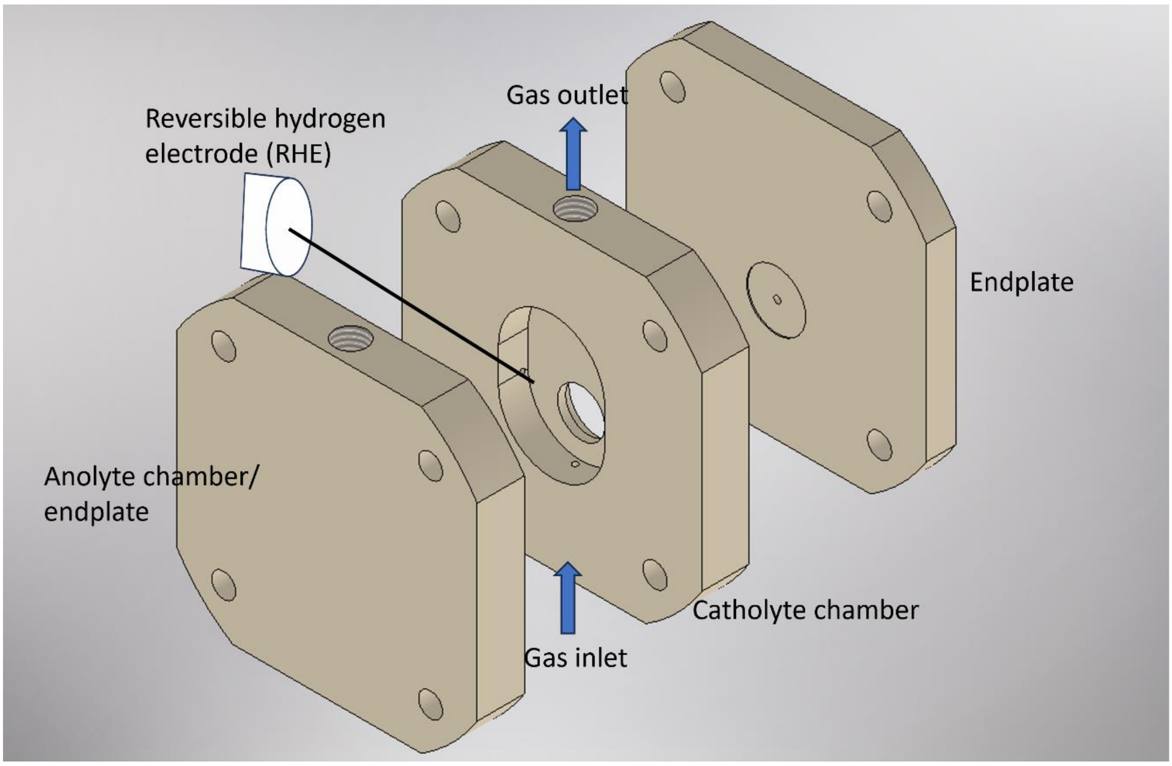

Fig. S1: Custom-made PEEK H-cell design used for all the experiments, sliced view with cathode chamber visible.

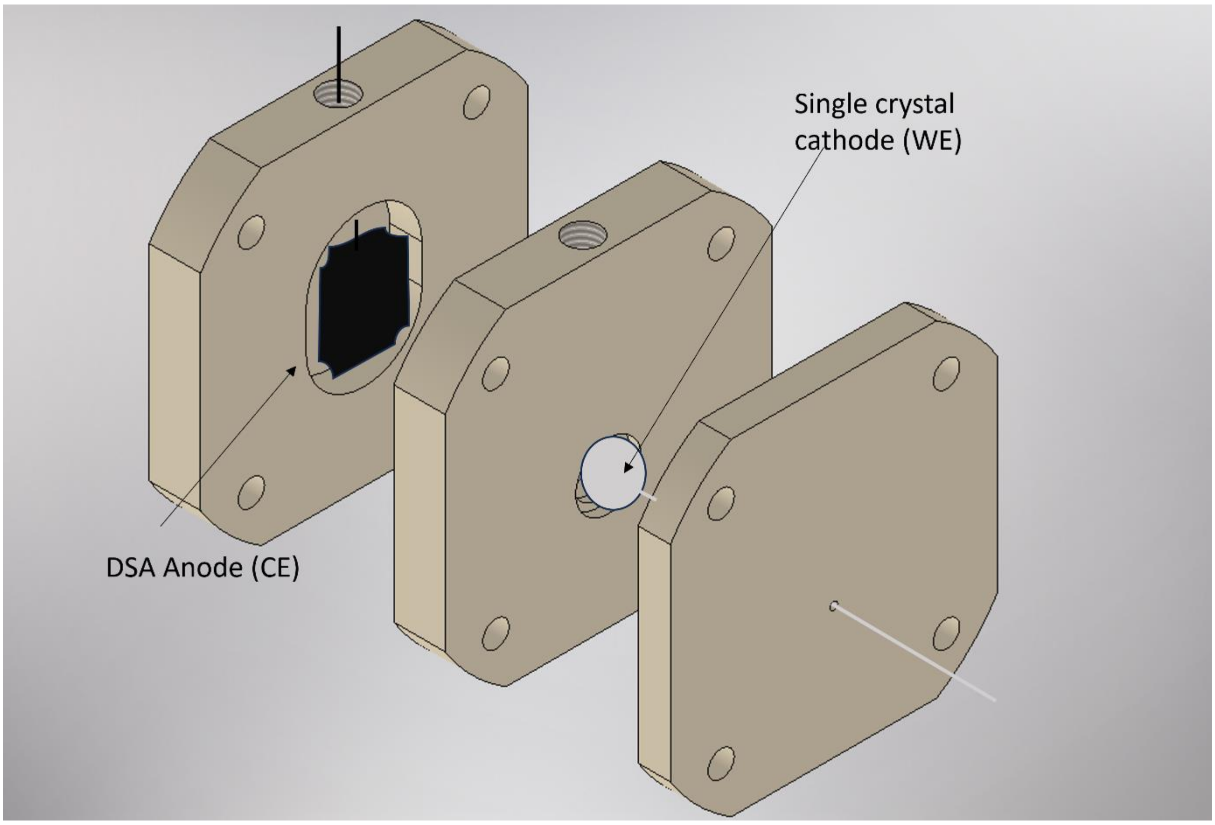

Fig. S2: Custom-made PEEK H-cell design used for all the experiments, sliced view with visible anode chamber, counter electrode and working electrode.

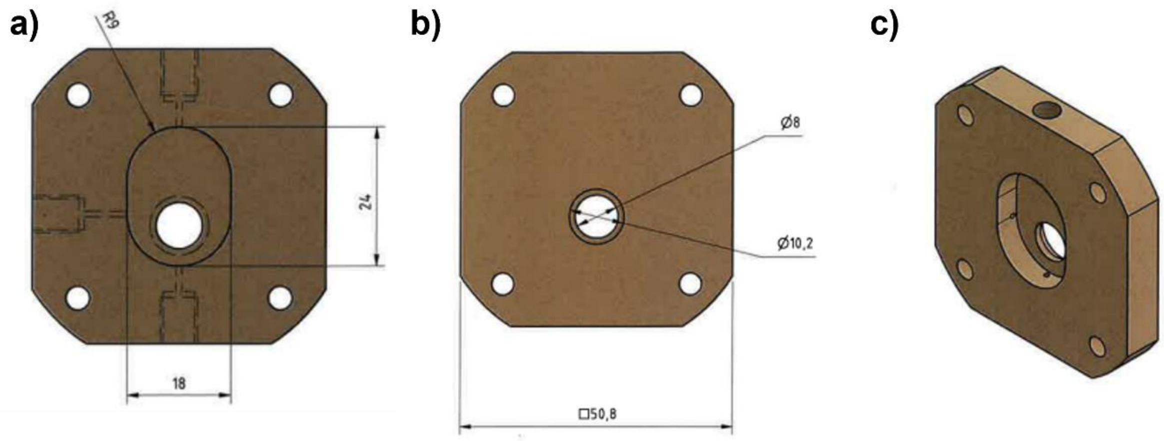

Fig. S3: A technical drawing of the custom H-cell with all dimensions in mm; a) the cathode chamber, b) the reverse side of the cathode chamber, with a single crystal insert; c) side view of the cathode chamber.

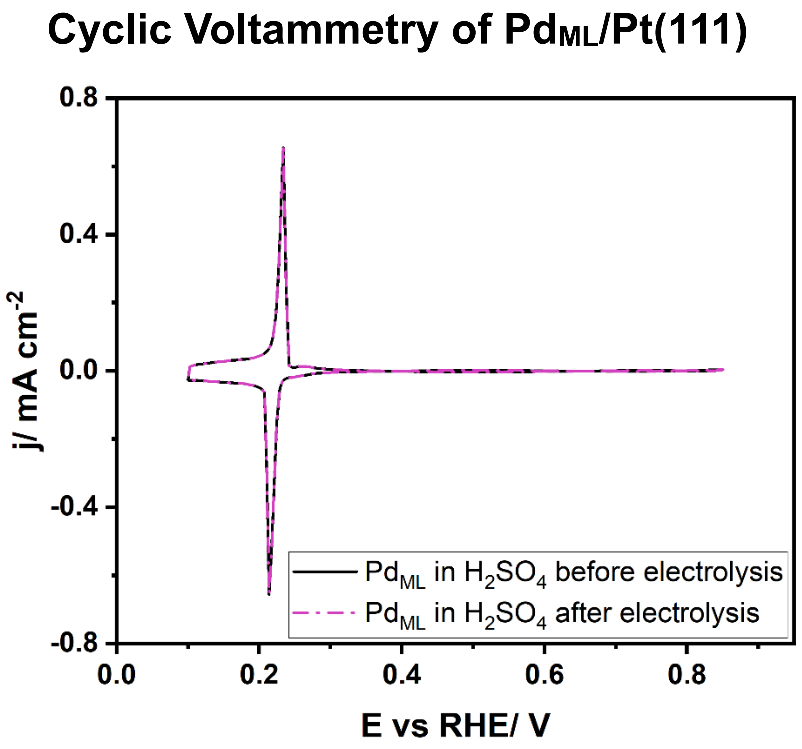

Fig. S4: Cyclic voltammetry (rate 50 mV/s) in 0.1 M H_2SO_4 of the Palladium monolayer before and after the electrolysis.

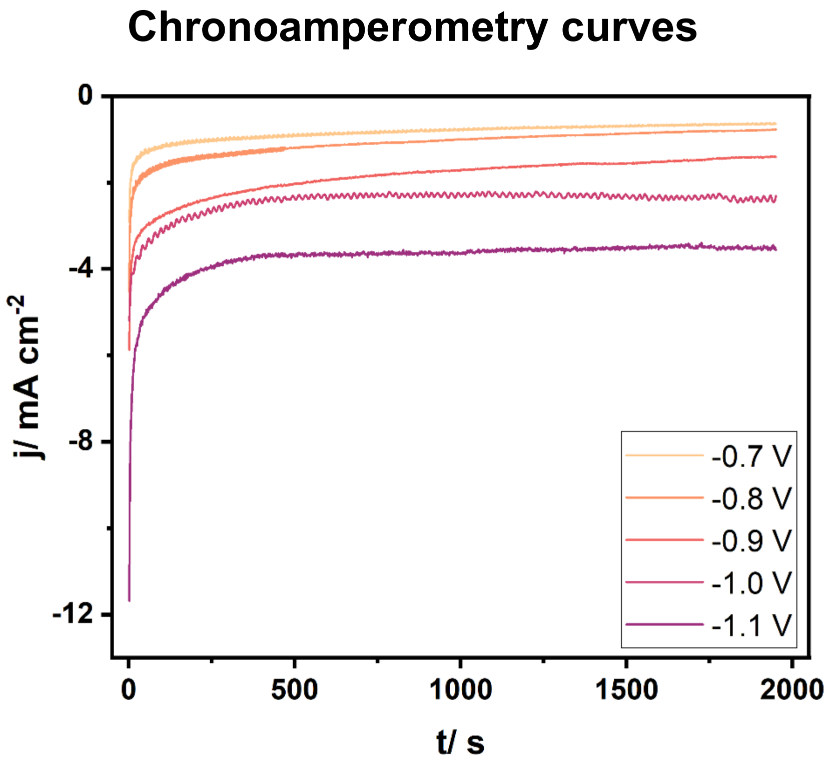

Fig. S5: Chronoamperometry results (normalized) for different electrolysis potentials.

NMR data

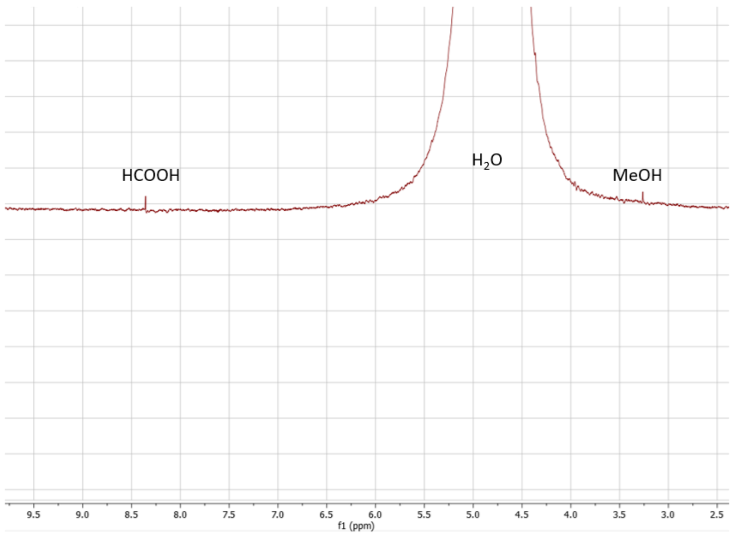

Fig. S6: ^{1}H-NMR of the liquid sample after CO_2RR at Pd_{ML} for 32 minutes at -0.8 V vs RHE. D_2O was used as a solvent to prepare the sample.

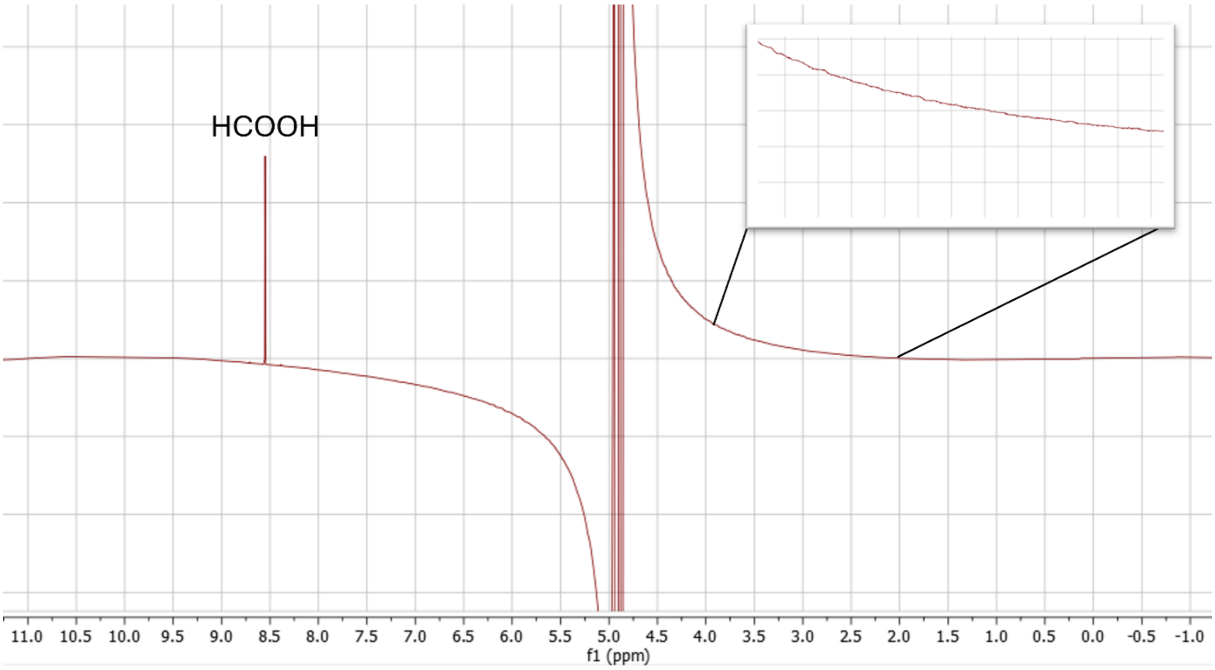

Fig. S7: ^{1}H-NMR of the liquid sample after HCOOH reduction at Pd_{ML} for 32 minutes at -0.7 V vs RHE. D_2O was used as a solvent to prepare the sample.

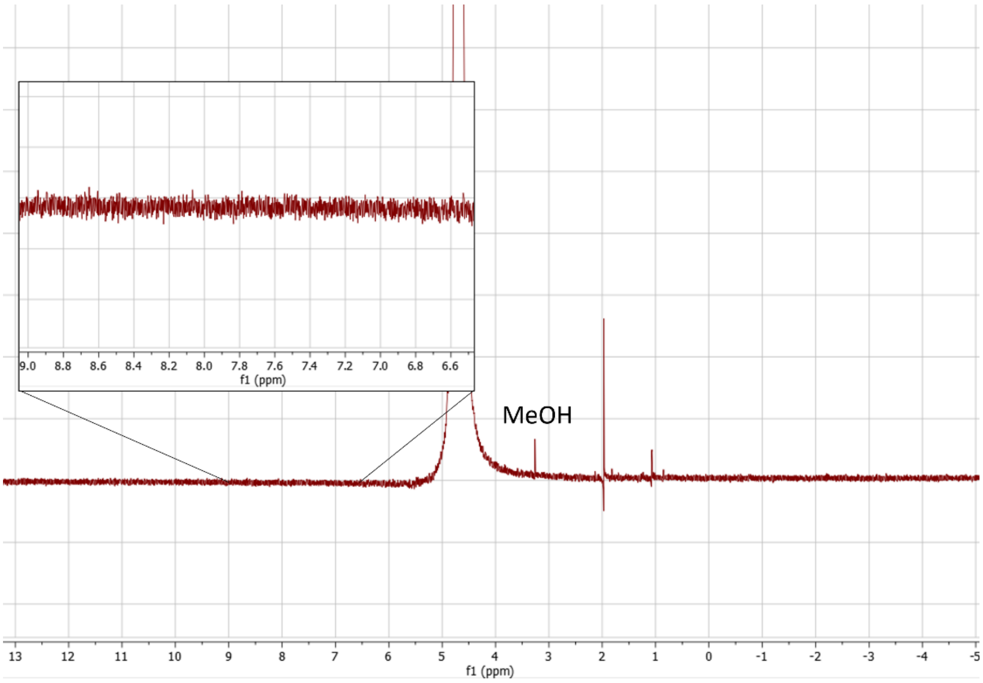

Fig. S8: ^{1}H-NMR of the liquid sample after CORR at Pd_{ML} for 32 minutes at -0.8 V vs RHE. D_2O was used as a solvent to prepare the sample.

HER data for $CO_{(2)}RR$ on Pd_{ML}

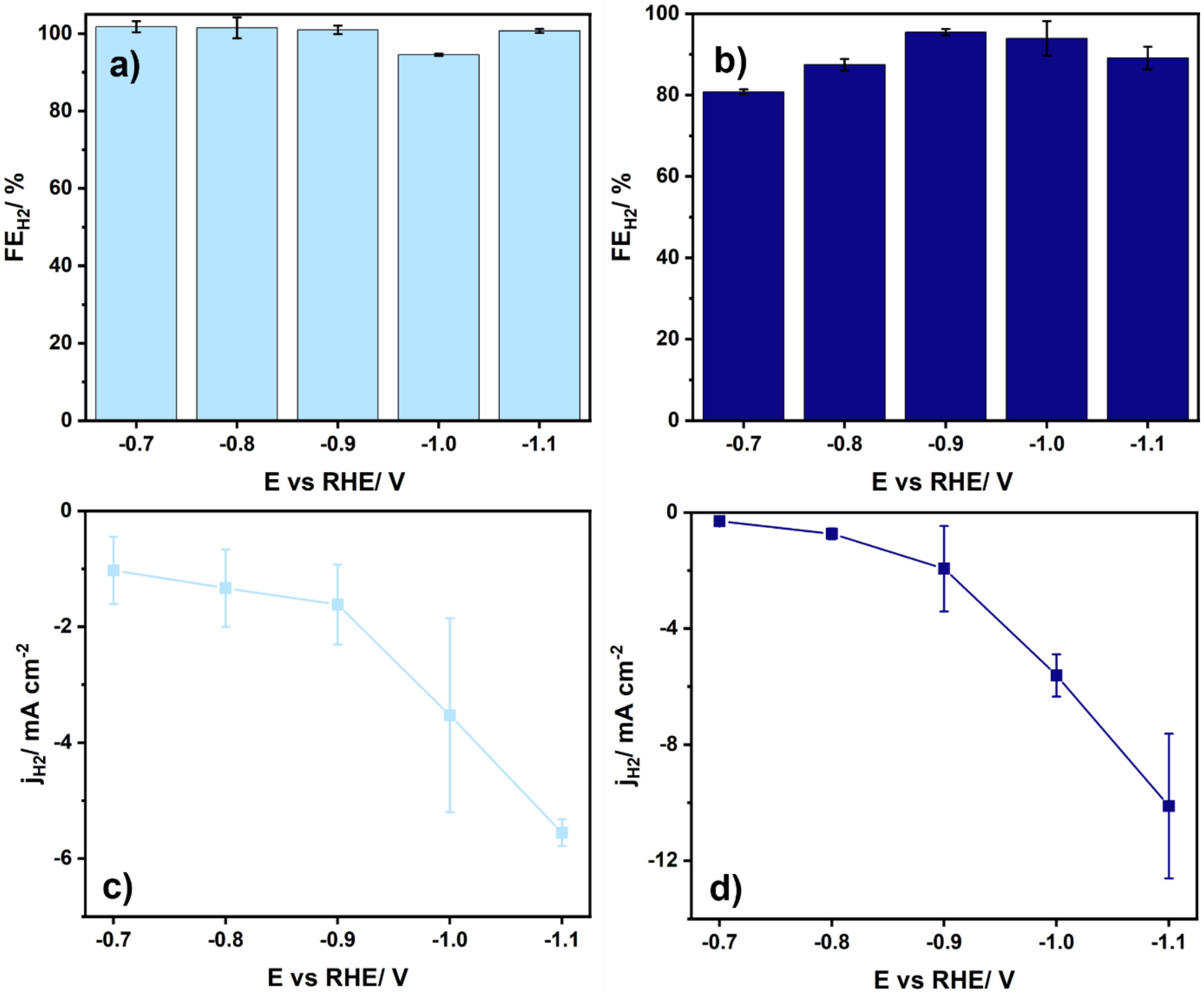

Fig. S9: a)-b) Faradaic efficiency towards hydrogen in CO_2 and CO reduction, respectively, and corresponding partial densities in c) CO_2RR and d) COR at Pd_{ML}/Pt(111).

HER data for HCHORR on Pd_{ML}

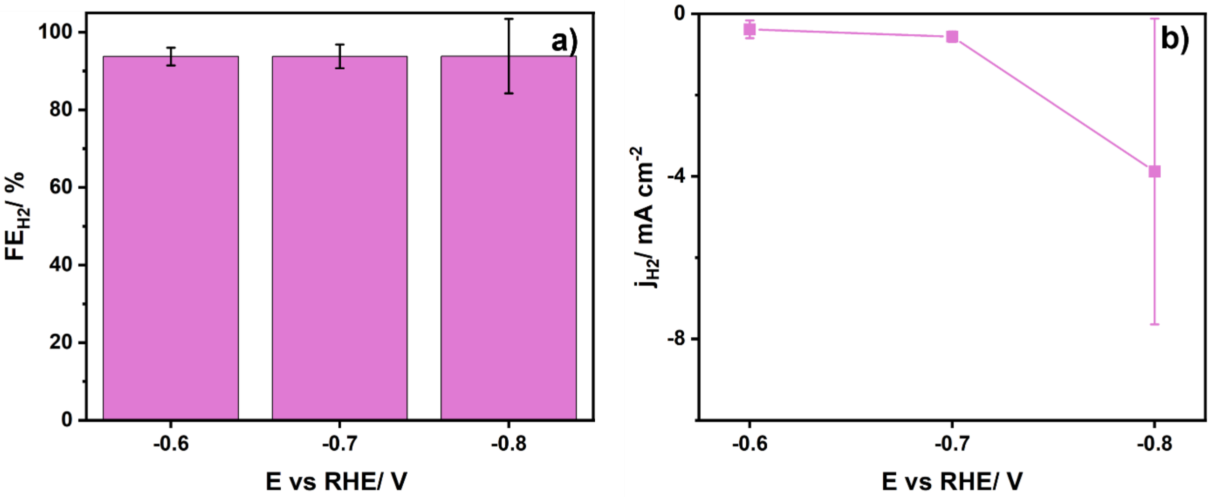

Fig. S10: a) Faradaic efficiency towards hydrogen in HCHO reduction on Pd_{ML}/Pt(111) and corresponding partial densities in b).

HER data for Pt(111)

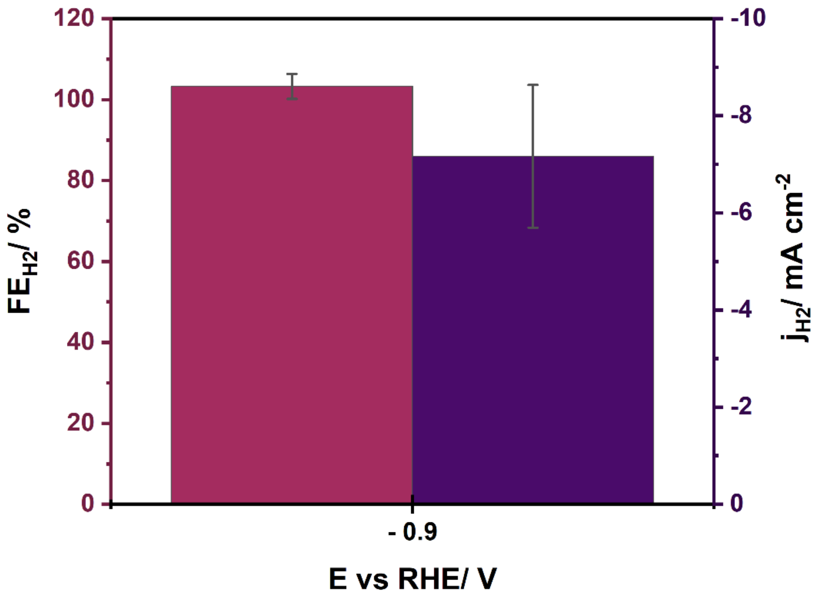

Fig. S11: Faradaic efficiency towards hydrogen and a corresponding partial density for CO_2RR at Pt(111) single crystal for 32 minutes at -0.9 V vs RHE.
